# Supplementary material for: Public Awareness and Behaviour in Great Britain in the Context of Sunlight Exposure and Vitamin D: Results from the First Large-Scale and Representative Survey
Source: Int J Environ Res Public Health. 2020 Sep 22;17(18):6924. doi: 10.3390/ijerph17186924 (PMC7557888; doi:10.3390/ijerph17186924)
Supplement: Supplementary file 1 [file ijerph-17-06924-s001.zip › ijerph-909532-supplementary/IJERPH909532_Supplementary material 2.docx]

Supplementary material 2: Skin colour of the survey respondents compared to ethnicity data in the 2011 UK Census of Population

**Table S1.** Skin colour of the survey respondents (*n* = 2024) compared to ethnicity data in the 2011 UK Census of Population.

| **Survey Respondents** | | **2011 UK Census** | |
| --- | --- | --- | --- |
| **Skin Colour** | **Percentage** | **Ethnicity** | **Percentage** |
| Types I–III: | 85% | White | 86% |
| Type IV–V | 10% | Asian | 8% |
| Type VI | 1% | Afro/Carib | 3% |
| Others | - | - | 3% |
| Don’t know | 3% | - | - |
| No response | 1% | - | - |
